# Supplementary material for: Leaf metabolic traits reveal hidden dimensions of plant form and function
Source: Sci Adv. 2023 Aug 30;9(35):eadi4029. doi: 10.1126/sciadv.adi4029 (PMC10468135; doi:10.1126/sciadv.adi4029)
Supplement: Supplementary file 1 — Figs. S1 to S5 Tables S1 to S4 References [file sciadv.adi4029_sm.pdf]

Science Advances
AAAS

Supplementary Materials for

Leaf metabolic traits reveal hidden dimensions of plant form and function

Tom W. N. Walker *et al.*

Corresponding author: Tom W. N. Walker, thomas.walker@unine.ch

Sci. Adv. **9**, eadi4029 (2023)
DOI: 10.1126/sciadv.adi4029

This PDF file includes:

Figs. S1 to S5
Tables S1 to S4
References

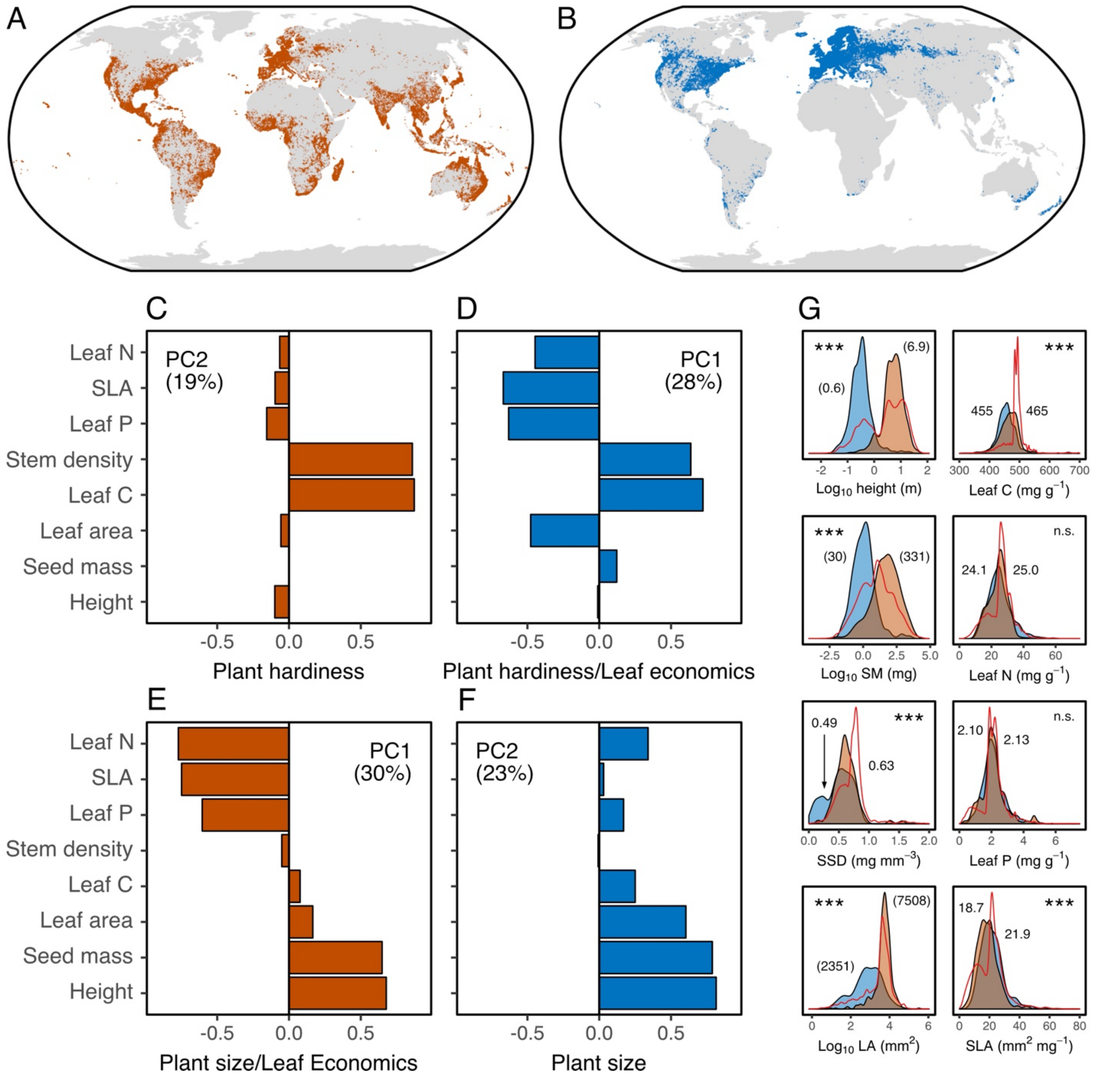

Supplementary Figure S1 | Tropical and temperate species capture major trade-offs in plant functional traits. (A,B) The global geographic extents of (A) tropical (brown; N = 457) and (B) temperate (blue; N = 405) plant species used in this study. Points are processed GBIF records representing native and non-native occurrences, with sample collection being restricted to *in situ* native populations. (C-F) Loadings for the first two PCs of PCAs performed on eight classical functional traits separately for (C,E) tropical and (D,F) temperate species. Axes collectively capture well-established life-history trade-offs (i.e., leaf economics spectrum (*16*), plant size/longevity (*7*, *104*), plant hardiness (*15*); see Main Text), although the coupling of these trade-offs and the ordering of PC axes differs between tropical and temperate species. (G) Kernel density estimates showing the distributions of classical traits used in PCAs (height, seed mass (SM), stem specific density (SSD), leaf area (LA) and leaf carbon, nitrogen and phosphorus) for tropical (blue) and temperate (brown) species, as well as for all species in the TRY database (red ribbon; (*92*)). Text shows group means, with values in brackets being back-transformed to original units ($\log_{10}$-transformed traits; see Methods). Asterisks illustrate significant differences ($P < 0.001$; linear models) between tropical and temperate species means.

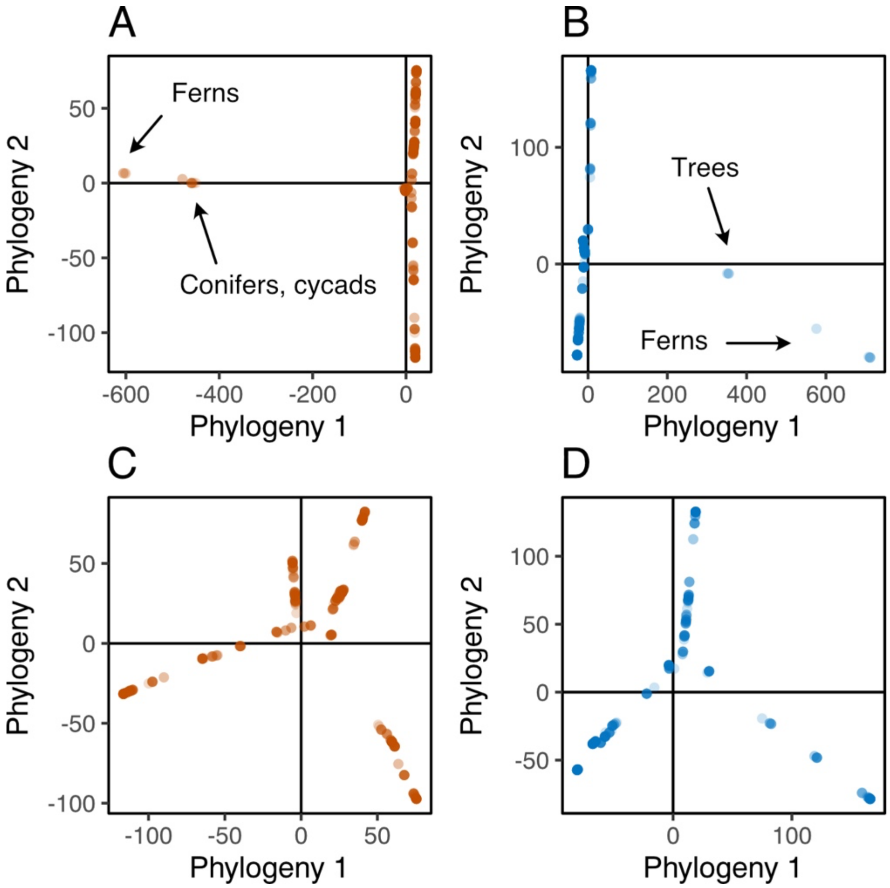

Supplementary Figure S2 | Ordinations of phylogenetic distances. Biplots of scores from the first two axes of principal coordinates analyses performed on cophenetic phylogenetic distances of (A,C) tropical (brown) and (B,D) temperate (blue) species, either (A,B) with (tropical N = 471, temperate N = 414) or (C,D) without (tropical N = 457, temperate N = 405) phylogenetically distinct species present. Subsetted data (C,D) were used for downstream analyses.

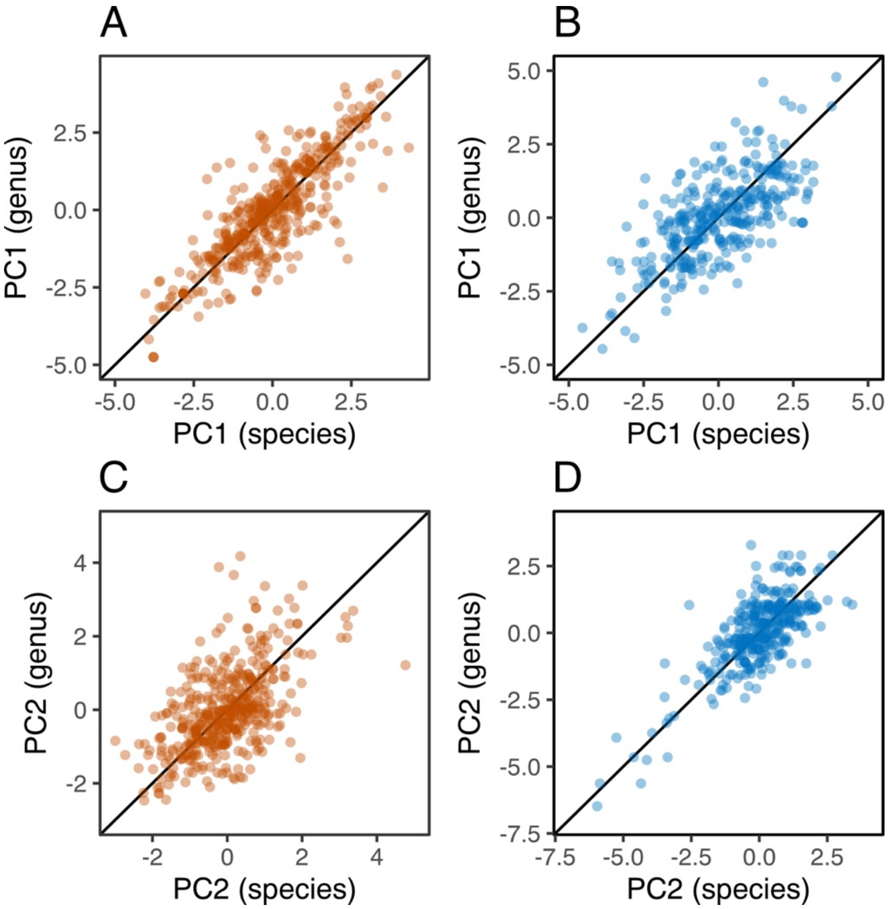

Supplementary Figure S3 | Using genus-level functional traits does not change nature of resulting PC axes. Correlations between (A,B) PC1 and (C,D) PC2 scores from PCAs performed on classical functional traits calculated at the species versus (x-axis) genus (y-axis) level separately for tropical (brown) and temperate (blue) species. A 1:1 line is displayed for reference.

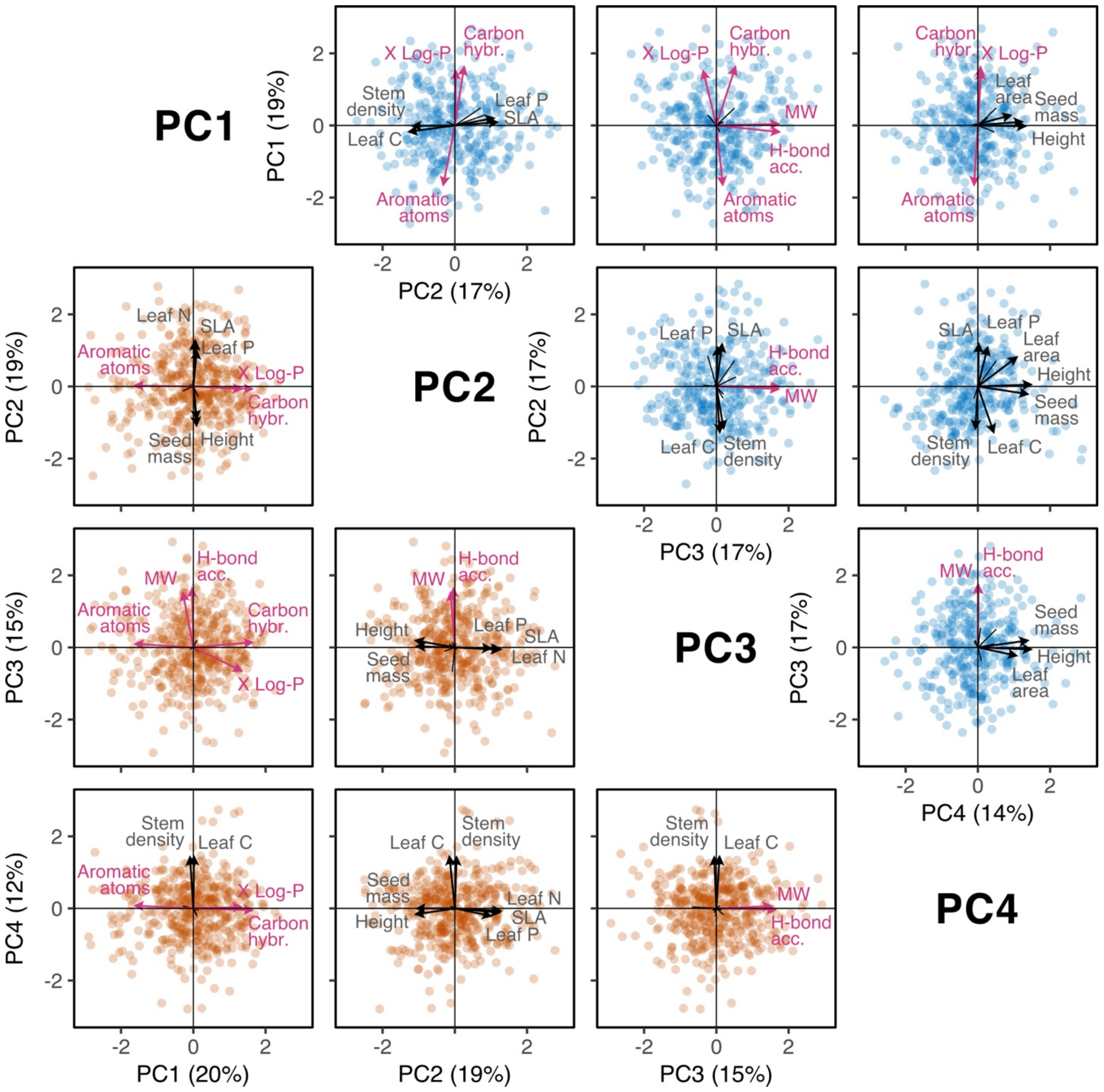

Supplementary Figure S4 | Metabolomic and classical functional traits are always orthogonal. A scatterplot matrix showing biplots of all combinations of the first four axes of PCAs performed on metabolomic (red) plus classical (grey) functional traits for tropical (bottom left, brown; N = 457) or temperate (top right, blue; N = 405) species. Points show positions of species on the two axes, while arrows show the strength and direction of trait loadings.

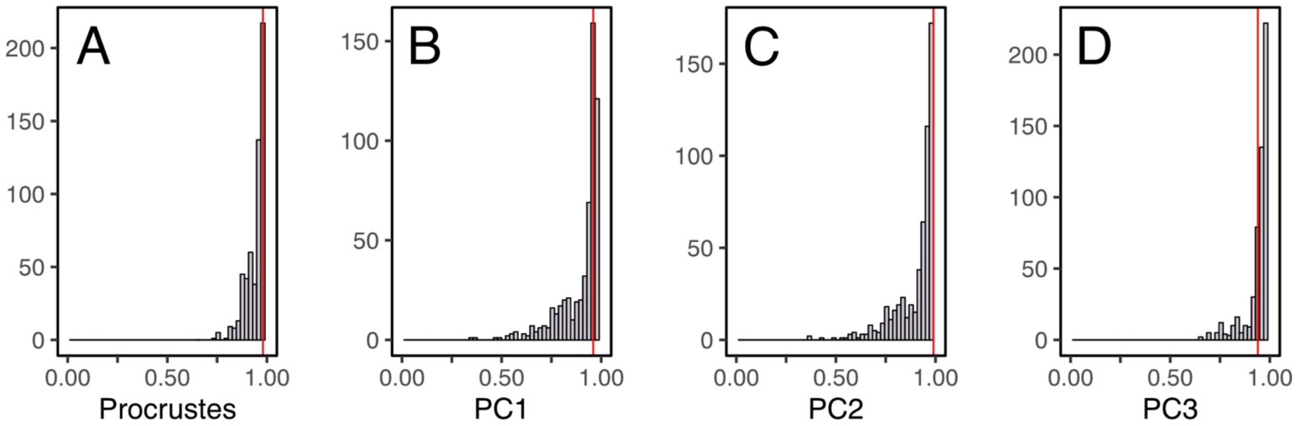

Supplementary Figure S5 | Subset PCAs are equivalent to the full PCA for most combinations of properties. Histograms of correlation coefficients between a full PCA containing all 21 chemical properties and subset PCAs performed on every combination of five chemical properties that cover the five clusters of leaf chemical variation (i.e. one from each cluster; N = 576). (A) Procrustes Rotation tests among contributing distance matrices. (B-D) Pairwise Pearson correlations among PC scores, where PCs from subset PCAs are first matched to corresponding PCs from the full PCA (i.e., allowing for the ordering of PCs to change among subset PCAs; *x*-axis). Red lines illustrate correlation coefficients for the subset PCA with selected chemical properties (see Text).

Table S1 | Selected chemical properties. Names, descriptions, and units of 21 quantitative chemical properties used to characterise leaf metabolite chemistry, as well as their observed ranges across all unique annotated metabolites from tropical and temperate species (see Main Text). Chemical properties were derived from the CDK (*27*) using SMILES chemical identifiers (Methods).

Category	Property	Description	Unit	Data range
Constitutional	Molecular weight (MW)	Total metabolite mass, as calculated from masses of constituent atoms	Da	80.1 – 3158.7
Constitutional	Total atom count	Total number of atoms	#	5 – 425
Constitutional	Aromatic atom count	Number of atoms in aromatic rings; high in shikimate pathway derivatives (e.g., flavonoids) (*105*)	#	0 – 36
Constitutional	Longest chain atom count	Number of atoms in longest chain; high in lipids (*106*)	#	3 – 53
Constitutional	Largest pi-system atom count	Number of atoms in largest conjugated system (i.e., alternating double-single bonds); high in pigments (*107*) and light-absorbing compounds (*108*)	#	0 – 42
Constitutional	Total bond count	Total number of (non-H) bonds	#	2 – 223
Constitutional	Aromatic bond count	Number of (non-H) bonds in aromatic rings; high in shikimate pathway derivatives (e.g., flavonoids) (*105*)	#	0 – 40
Constitutional	Rotatable bond count	Number of rotatable bonds (i.e., single, non-ring, non-terminal bonds with low energy barrier for rotation); negatively correlates with passive transport across biological membranes (*109*)	#	0 – 86
Constitutional	X log *P*	Octanol/water partition coefficient calculated using a modified atom-additive model summing atomic contributions and correcting for intramolecular interactions (*47*); positive indicates affinity to octanol (i.e., nonpolar, hydrophobic), negative to water (i.e., polar, hydrophilic)	Log-ratio	-12.7 – 32.1
Constitutional	A log *P*	Octanol/water partition coefficient calculated using an atom-additive model summing atom type contributions based on focal atom and bond characteristics (*48*); positive indicates affinity to octanol (i.e., nonpolar, hydrophobic), negative to water (i.e., polar, hydrophilic)	Log-ratio	-15.1 – 27.2
Constitutional	M log *P*	Octanol/water partition coefficient calculated using a simple equation dependent on the number of C atoms and number of hetero atoms (*38*); smaller indicates affinity to octanol (i.e., nonpolar, hydrophobic), larger to water (i.e., polar, hydrophilic)	Log-ratio	0.7 – 8.3
Topological	Topological polar surface area (PSA)	Sum of surface area of polar atoms, calculated from contributions of polar molecular fragments (*110*); in pharmaceutical studies, PSA correlates inversely with passive transport through biological membranes (lower TPSA means greater fraction absorbed) (*111*)	Å	0 – 1468.2
Topological	MW-specific PSA	PSA divided by metabolite molecular mass; in pharmaceutical studies, PSA correlates inversely with passive transport through biological membranes (*111*)	Å Da^{-1}	0 – 0.77
Topological	Hybridisation ratio	Fraction of sp_3 to sp_2 carbon atoms; proxy for bond saturation and three-dimensional topological complexity; positive correlate of melting point, solubility, and bioactivity (*30*)	Ratio	0 – 1
Topological	Fractional CSP_3	Fraction of sp_3 carbon atoms to total carbon count; proxy for bond saturation and three-dimensional topological complexity; positive correlate of melting point, solubility, and bioactivity (*30*)	Ratio	0 – 1
Topological	f_{MF}	Ratio between size of molecular framework (ring atoms plus linkers) and size of metabolite; complexity measure positively correlated to promiscuity (number of protein targets ≥50% inhibited) at values above 0.65 (*41*)	Ratio	0 – 1
Topological	Eccentric connectivity index	Distance-cum-adjacency topological descriptor (higher for longer chains with less branching); correlates with size and physicochemical properties (e.g., boiling point) (*40*)	-	6 – 14213
Topological	Wiener path number	Topological descriptor of molecular branching that can differentiate structural isomers; correlates positively with size and boiling point (*39*)	#	4 – 413341
Topological	Wiener polarity number	A variant of Wiener path number calculated using vertices (C atoms) at distance 3; correlates positively with size and boiling point (*39*)	#	0 – 354
Electronic	H-bond donor count	Number of H-bond donors (OH/NH, formal charge ≥ 0); H-bonds are intermolecular forces essential for macromolecules and complexes (*112*)	#	0 – 44
Electronic	H-bond acceptor count	Number of H-bond acceptors (O/N, formal charge ≤ 0, non-ether O, non-adjacent ON); H-bonds are intermolecular forces essential for macromolecules and complexes (*112*)	#	0 – 75

Table S2 | Relative differences between the sizes of hypervolumes for four null models and those observed for metabolic or classical functional traits. Percent differences between mean null model hypervolume sizes (999 permutations) versus observed hypervolumes for metabolic (MT) or classical (FT) functional traits. Values not in parentheses are statistically significant (underlined: $P < 0.05$; others: $P < 0.01$).

**Null model**		**Tropical species**		**Temperate species**	
Trait distribution	Trait covariance	MTs	FTs	MTs	FTs
Uniform	Independent	- 99.8%	- 99.9%	- 99.8%	- 99.5%
Normal	Independent	- 98.2%	- 52.7%	- 98.1%	- 56.6%
As observed	Independent	- 98.3%	- 48.9%	- 98.1%	- 53.7%
Normal	As observed	+ 27.7%	(- 6.5%)	+ 15.3%	- 15.2%

Table S3 | Relative differences between the lumpiness of hypervolumes for four null models and those observed for metabolic or classical functional traits. Mean percent differences between the minimum number of cells in multidimensional space needed to cover 10% species for four null model hypervolumes (999 permutations) versus observed hypervolumes for metabolic (MTs) or classical (FT) functional traits. Values not in parentheses are statistically significant ($P < 0.01$).

**Null model**		**Tropical species**		**Temperate species**	
Trait distribution	Trait covariance	MTs	FTs	MTs	FTs
Uniform	Independent	- 94.7%	- 44.1%	- 89.2%	- 26.4%
Normal	Independent	- 83.6%	- 65.2%	- 71.4%	- 22.6%
As observed	Independent	- 81.6%	- 57.2%	- 71.0%	- 19.2%
Normal	As observed	(- 15.8%)	- 62.7%	(+ 5.2%)	- 18.9%

Table S4 | Classical functional trait coverage. Percent coverage for the eight classical functional traits used here in the original TRY dataset and following 90 iterations of BHPMF imputations. In both cases, clear outliers were removed (see Methods).

**Trait**	**Tropical species**		**Temperate species**	
	**No imputation**	**BHPMF**	**No imputation**	**BHPMF**
Plant height	41.6%	81.6%	99.7%	99.7%
Seed mass	67.2%	87.1%	92.0%	92.3%
Stem density	50.3%	58.2%	17.7%	27.4%
Leaf area	45.7%	69.1%	88.2%	89.7%
Specific leaf area	48.1%	57.1%	91.2%	94.4%
Leaf carbon content	32.8%	46.8%	75.8%	80.2%
Leaf nitrogen content	45.3%	63.2%	79.9%	91.4%
Leaf phosphorus content	40.7%	62.6%	56.0%	94.1%

REFERENCES AND NOTES

1. S. Wang, S. Alseekh, A. R. Fernie, J. Luo, The structure and function of major plant metabolite modifications. *Mol. Plant* **12**, 899–919 (2019).

2. G. Faccio, Plant complexity and cosmetic innovation. *iScience* **23**, 101358 (2020).

3. H. J. Klee, D. M. Tieman, The genetics of fruit flavour preferences. *Nat. Rev. Genet.* **19**, 347–356 (2018).

4. B. M. Schmidt, D. M. Ribnicky, P. E. Lipsky, I. Raskin, Revisiting the ancient concept of botanical therapeutics. *Nat. Chem. Biol.* **3**, 360–366 (2007).

5. J. Sardans, A. Gargallo-Garriga, O. Urban, K. Klem, T. W. N. Walker, P. Holub, I. A. Janssens, J. Peñuelas, Ecometabolomics for a better understanding of plant responses and acclimation to abiotic factors linked to global change. *Metabolites* **10**, 239 (2020).

6. J. Sardans, A. Gargallo-Garriga, O. Urban, K. Klem, P. Holub, I. A. Janssens, T. W. N. Walker, A. Pesqueda, J. Peñuelas, Ecometabolomics of plant–herbivore and plant–fungi interactions: A synthesis study. *Ecosphere* **12**, e03736 (2021).

7. S. Díaz, J. Kattge, J. H. C. Cornelissen, I. J. Wright, S. Lavorel, S. Dray, B. Reu, M. Kleyer, C. Wirth, I. C. Prentice, E. Garnier, G. Bönisch, M. Westoby, H. Poorter, P. B. Reich, A. T. Moles, J. Dickie, A. N. Gillison, A. E. Zanne, J. Chave, S. J. Wright, S. N. Sheremet'ev, H. Jactel, C. Baraloto, B. Cerabolini, S. Pierce, B. Shipley, D. Kirkup, F. Casanoves, J. S. Joswig, A. Günther, V. Falczuk, N. Rüger, M. D. Mahecha, L. D. Gorné, The global spectrum of plant form and function. *Nature* **529**, 167–171 (2016).

8. M. Wink, Evolution of secondary metabolites from an ecological and molecular phylogenetic perspective. *Phytochemistry* **64**, 3–19 (2003).

9. M. C. Schuman, N. M. van Dam, F. Beran, W. S. Harpole, How does plant chemical diversity contribute to biodiversity at higher trophic levels? *Curr. Opin. Insect Sci.* **14**, 46–55 (2016).

10. A. E. Zanne, D. C. Tank, W. K. Cornwell, J. M. Eastman, S. A. Smith, R. G. FitzJohn, D. J. McGlinn, B. C. O'Meara, A. T. Moles, P. B. Reich, D. L. Royer, D. E. Soltis, P. F. Stevens, M. Westoby, I. J. Wright, L. Aarssen, R. I. Bertin, A. Calaminus, R. Govaerts, F. Hemmings, M. R. Leishman, J. Oleksyn, P. S. Soltis, N. G. Swenson, L. Warman, J. M. Beaulieu, Three keys to the radiation of angiosperms into freezing environments. *Nature* **506**, 89–92 (2014).

11. G. E. Hutchinson, *The Ecological Theater and the Evolutionary Play* (Yale Univ. Press, 1965).

12. P. Feeny, in *Biochemical Interaction Between Plants and Insects*, J. W. Wallace, R. L. Mansell, Eds. (Springer, 1976), pp. 1–40.

13. P. D. Coley, J. P. Bryant, F. S. Chapin, Resource availability and plant antiherbivore defense. *Science* **230**, 895–899 (1985).

14. P. Capdevila, T. W. N. Walker, F. Schrodt, R. C. Rodriguez Caro, R. Salguero-Gomez, Global patterns of plant form and function are strongly determined by evolutionary relationships. *bioRxiv*, 2023.01.13.523963 (2023).

15. J. P. Grime, K. Thompson, R. Hunt, J. G. Hodgson, J. H. C. Cornelissen, I. H. Rorison, G. A. F. Hendry, T. W. Ashenden, A. P. Askew, S. R. Band, R. E. Booth, C. C. Bossard, B. D. Campbell, J. E. L. Cooper, A. W. Davison, P. L. Gupta, W. Hall, D. W. Hand, M. A. Hannah, S. H. Hillier, D. J. Hodkinson, A. Jalili, Z. Liu, J. M. L. Mackey, N. Matthews, M. A. Mowforth, A. M. Neal, R. J. Reader, K. Reiling, W. Ross-Fraser, R. E. Spencer, F. Sutton, D. E. Tasker, P. C. Thorpe, J. Whitehouse, Integrated screening validates primary axes of specialisation in plants. *Oikos* **79**, 259 (1997).

16. I. J. Wright, P. B. Reich, M. Westoby, D. D. Ackerly, Z. Baruch, F. Bongers, J. Cavender-Bares, T. Chapin, J. H. C. Cornelissen, M. Diemer, J. Flexas, E. Garnier, P. K. Groom, J. Gulias, K. Hikosaka, B. B. Lamont, T. Lee, W. Lee, C. Lusk, J. J. Midgley, M.-L. Navas, U. Niinemets, J. Oleksyn, N. Osada, H. Poorter, P. Poot, L. Prior, V. I. Pyankov, C. Roumet, S. C. Thomas, M. G. Tjoelker, E. J. Veneklaas, R. Villar, The worldwide leaf economics spectrum. *Nature* **428**, 821–827 (2004).

17. T. W. N. Walker, J. M. Alexander, P. Allard, O. Baines, V. Baldy, R. D. Bardgett, P. Capdevila, P. D. Coley, B. David, E. Defossez, M. Endara, M. Ernst, C. Fernandez, D. Forrister, A. Gargallo-Garriga, V.

E. J. Jassey, S. Marr, S. Neumann, L. Pellissier, J. Peñuelas, K. Peters, S. Rasmann, U. Roessner, J. Sardans, F. Schrodt, M. C. Schuman, A. Soule, H. Uthe, W. Weckwerth, J. Wolfender, N. M. Dam, R. Salguero-Gómez, Functional Traits 2.0: The power of the metabolome for ecology. *J. Ecol.* **110**, 4–20 (2022).

18. B. E. Sedio, J. D. Parker, S. M. McMahon, S. J. Wright, Comparative foliar metabolomics of a tropical and a temperate forest community. *Ecology* **99**, 2647–2653 (2018).

19. D. L. Forrister, M.-J. Endara, G. C. Younkin, P. D. Coley, T. A. Kursar, Herbivores as drivers of negative density dependence in tropical forest saplings. *Science* **363**, 1213–1216 (2019).

20. A. Gargallo-Garriga, J. Sardans, V. Granda, J. Llusià, G. Peguero, D. Asensio, R. Ogaya, I. Urbina, L. Van Langenhove, L. T. Verryckt, J. Chave, E. A. Courtois, C. Stahl, O. Grau, K. Klem, O. Urban, I. A. Janssens, J. Peñuelas, Different "metabolomic niches" of the highly diverse tree species of the French Guiana rainforests. *Sci. Rep.* **10**, 6937 (2020).

21. C. Scherling, C. Roscher, P. Giavalisco, E.-D. Schulze, W. Weckwerth, Metabolomics unravel contrasting effects of biodiversity on the performance of individual plant species. *PLOS ONE* **5**, e12569 (2010).

22. C. Violle, M.-L. Navas, D. Vile, E. Kazakou, C. Fortunel, I. Hummel, E. Garnier, Let the concept of trait be functional! *Oikos* **116**, 882–892 (2007).

23. P. B. Adler, R. Salguero-Gómez, A. Compagnoni, J. S. Hsu, J. Ray-Mukherjee, C. Mbeau-Ache, M. Franco, Functional traits explain variation in plant life history strategies. *Proc. Natl. Acad. Sci. U.S.A.* **111**, 740–745 (2014).

24. H. Bruelheide, J. Dengler, O. Purschke, J. Lenoir, B. Jiménez-Alfaro, S. M. Hennekens, Z. Botta-Dukát, M. Chytrý, R. Field, F. Jansen, J. Kattge, V. D. Pillar, F. Schrodt, M. D. Mahecha, R. K. Peet, B. Sandel, P. van Bodegom, J. Altman, E. Alvarez-Dávila, M. A. S. Arfin Khan, F. Attorre, I. Aubin, C. Baraloto, J. G. Barroso, M. Bauters, E. Bergmeier, I. Biurrun, A. D. Bjorkman, B. Blonder, A. Čarni, L. Cayuela, T. Černý, J. H. C. Cornelissen, D. Craven, M. Dainese, G. Derroire, M. De Sanctis, S. Díaz, J. Doležal, W. Farfan-Rios, T. R. Feldpausch, N. J. Fenton, E. Garnier, G. R. Guerin, A. G. Gutiérrez, S.

Haider, T. Hattab, G. Henry, B. Hérault, P. Higuchi, N. Hölzel, J. Homeier, A. Jentsch, N. Jürgens, Z. Kącki, D. N. Karger, M. Kessler, M. Kleyer, I. Knollová, A. Y. Korolyuk, I. Kühn, D. C. Laughlin, F. Lens, J. Loos, F. Louault, M. I. Lyubenova, Y. Malhi, C. Marcenò, M. Mencuccini, J. V. Müller, J. Munzinger, I. H. Myers-Smith, D. A. Neill, Ü. Niinemets, K. H. Orwin, W. A. Ozinga, J. Penuelas, A. Pérez-Haase, P. Petřík, O. L. Phillips, M. Pärtel, P. B. Reich, C. Römermann, A. V. Rodrigues, F. M. Sabatini, J. Sardans, M. Schmidt, G. Seidler, J. E. Silva Espejo, M. Silveira, A. Smyth, M. Sporbert, J.-C. Svenning, Z. Tang, R. Thomas, I. Tsiripidis, K. Vassilev, C. Violle, U. Jandt, Global trait-environment relationships of plant communities. *Nat. Ecol. Evol.* **2**, 1906–1917 (2018).

25. G. B. De Deyn, J. H. C. Cornelissen, R. D. Bardgett, Plant functional traits and soil carbon sequestration in contrasting biomes. *Ecol. Lett.* **11**, 516–531 (2008).

26. S. Lavorel, J. Storkey, R. D. Bardgett, F. de Bello, M. P. Berg, X. Le Roux, M. Moretti, C. Mulder, R. J. Pakeman, S. Díaz, R. Harrington, A novel framework for linking functional diversity of plants with other trophic levels for the quantification of ecosystem services. *J. Veg. Sci.* **24**, 942–948 (2013).

27. E. L. Willighagen, J. W. Mayfield, J. Alvarsson, A. Berg, L. Carlsson, N. Jeliazkova, S. Kuhn, T. Pluskal, M. Rojas-Chertó, O. Spjuth, G. Torrance, C. T. Evelo, R. Guha, C. Steinbeck, The Chemistry Development Kit (CDK) v2.0: Atom typing, depiction, molecular formulas, and substructure searching. *J. Chem.* **9**, 33 (2017).

28. J. Rosén, A. Lövgren, T. Kogej, S. Muresan, J. Gottfries, A. Backlund, ChemGPS-NP(Web): Chemical space navigation online. *J. Comput. Aided Mol. Des.* **23**, 253–259 (2009).

29. C. A. Lipinski, F. Lombardo, B. W. Dominy, P. J. Feeney, Experimental and computational approaches to estimate solubility and permeability in drug discovery and development settings. *Adv. Drug Deliv. Rev.* **46**, 3–26 (2001).

30. F. Lovering, J. Bikker, C. Humblet, Escape from flatland: Increasing saturation as an approach to improving clinical success. *J. Med. Chem.* **52**, 6752–6756 (2009).

31. J. Larsson, J. Gottfries, S. Muresan, A. Backlund, ChemGPS-NP: Tuned for navigation in biologically relevant chemical space. *J. Nat. Prod.* **70**, 789–794 (2007).

32. J. L. Funk, J. E. Larson, G. M. Ames, B. J. Butterfield, J. Cavender-Bares, J. Firn, D. C. Laughlin, A. E. Sutton-Grier, L. Williams, J. Wright, Revisiting the Holy Grail: Using plant functional traits to understand ecological processes. *Biol. Rev. Camb. Philos. Soc.* **92**, 1156–1173 (2017).

33. J. Firn, J. M. McGree, E. Harvey, H. Flores-Moreno, M. Schütz, Y. M. Buckley, E. T. Borer, E. W. Seabloom, K. J. La Pierre, A. M. MacDougall, S. M. Prober, C. J. Stevens, L. L. Sullivan, E. Porter, E. Ladouceur, C. Allen, K. H. Moromizato, J. W. Morgan, W. S. Harpole, Y. Hautier, N. Eisenhauer, J. P. Wright, P. B. Adler, C. A. Arnillas, J. D. Bakker, L. Biederman, A. A. D. Broadbent, C. S. Brown, M. N. Bugalho, M. C. Caldeira, E. E. Cleland, A. Ebeling, P. A. Fay, N. Hagenah, A. R. Kleinhesselink, R. Mitchell, J. L. Moore, C. Nogueira, P. L. Peri, C. Roscher, M. D. Smith, P. D. Wragg, A. C. Risch, Leaf nutrients, not specific leaf area, are consistent indicators of elevated nutrient inputs. *Nat. Ecol. Evol.* **3**, 400–406 (2019).

34. F. van der Plas, T. Schröder-Georgi, A. Weigelt, K. Barry, S. Meyer, A. Alzate, R. L. Barnard, N. Buchmann, H. de Kroon, A. Ebeling, N. Eisenhauer, C. Engels, M. Fischer, G. Gleixner, A. Hildebrandt, E. Koller-France, S. Leimer, A. Milcu, L. Mommer, P. A. Niklaus, Y. Oelmann, C. Roscher, C. Scherber, M. Scherer-Lorenzen, S. Scheu, B. Schmid, E.-D. Schulze, V. Temperton, T. Tscharntke, W. Voigt, W. Weisser, W. Wilcke, C. Wirth, Plant traits alone are poor predictors of ecosystem properties and long-term ecosystem functioning. *Nat. Ecol. Evol.* **4**, 1602–1611 (2020).

35. T. W. N. Walker, W. Weckwerth, L. Bragazza, L. Fragner, B. G. Forde, N. J. Ostle, C. Signarbieux, X. Sun, S. E. Ward, R. D. Bardgett, Plastic and genetic responses of a common sedge to warming have contrasting effects on carbon cycle processes. *Ecol. Lett.* **22**, 159–169 (2019).

36. P.-M. Allard, A. Gaudry, L.-M. Quirós-Guerrero, A. Rutz, M. Dounoue-Kubo, T. W. N. Walker, E. Defossez, C. Long, A. Grondin, B. David, J.-L. Wolfender, Open and reusable annotated mass spectrometry dataset of a chemodiverse collection of 1,600 plant extracts. *Gigascience* **12**, giac124 (2022).

37. E. Defossez, C. Pitteloud, P. Descombes, G. Glauser, P.-M. Allard, T. W. N. Walker, P. Fernandez-Conradi, J.-L. Wolfender, L. Pellissier, S. Rasmann, Spatial and evolutionary predictability of phytochemical diversity. *Proc. Natl. Acad. Sci. U.S.A.* **118**, e2013344118 (2021).

38. R. Mannhold, G. I. Poda, C. Ostermann, I. V. Tetko, Calculation of molecular lipophilicity: State-of-the-art and comparison of log P methods on more than 96,000 compounds. *J. Pharm. Sci.* **98**, 861–893 (2009).

39. H. Wiener, Structural determination of paraffin boiling points. *J. Am. Chem. Soc.* **69**, 17–20 (1947).

40. V. Sharma, R. Goswami, A. K. Madan, Eccentric connectivity index: A novel highly discriminating topological descriptor for structure−property and structure−activity studies. *J. Chem. Inf. Comput. Sci.* **37**, 273–282 (1997).

41. Y. Yang, H. Chen, I. Nilsson, S. Muresan, O. Engkvist, Investigation of the relationship between topology and selectivity for druglike molecules. *J. Med. Chem.* **53**, 7709–7714 (2010).

42. B. Kuhn, P. Mohr, M. Stahl, Intramolecular hydrogen bonding in medicinal chemistry. *J. Med. Chem.* **53**, 2601–2611 (2010).

43. Th. Zeegers-Huyskens, P. Huyskens, in *Intermolecular Forces*, P. L. Huyskens, W. A. P. Luck, T. Zeegers-Huyskens, Eds. (Springer Berlin Heidelberg, Berlin, Heidelberg, 1991), pp. 1–30.

44. P. B. Dervan, Molecular recognition of DNA by small molecules. *Bioorg. Med. Chem.* **9**, 2215–2235 (2001).

45. G. Padroni, N. N. Patwardhan, M. Schapira, A. E. Hargrove, Systematic analysis of the interactions driving small molecule-RNA recognition. *RSC Med. Chem.* **11**, 802–813 (2020).

46. M. Gao, J. Skolnick, A comprehensive survey of small-molecule binding pockets in proteins. *PLoS Comput. Biol.* **9**, e1003302 (2013).

47. R. Wang, Y. Fu, L. Lai, A new atom-additive method for calculating partition coefficients. *J. Chem. Inf. Comput. Sci.* **37**, 615–621 (1997).

48. A. K. Ghose, G. M. Crippen, Atomic physicochemical parameters for three-dimensional structure-directed quantitative structure-activity relationships I. Partition coefficients as a measure of hydrophobicity. *J. Comput. Chem.* **7**, 565–577 (1986).

49. P. A. Clemons, N. E. Bodycombe, H. A. Carrinski, J. A. Wilson, A. F. Shamji, B. K. Wagner, A. N. Koehler, S. L. Schreiber, Small molecules of different origins have distinct distributions of structural complexity that correlate with protein-binding profiles. *Proc. Natl. Acad. Sci. U.S.A.* **107**, 18787–18792 (2010).

50. J. N. Israelachvili, The nature of van der waals forces. *Contemp. Phys.* **15**, 159–178 (1974).

51. D. Van Vranken, G. A. Weiss, *Introduction to Bioorganic Chemistry and Chemical Biology* (Garland Science, ed. 1, 2012).

52. E. Pichersky, R. A. Raguso, Why do plants produce so many terpenoid compounds? *New Phytol.* **220**, 692–702 (2018).

53. D. L. Forrister, M.-J. Endara, A. J. Soule, G. C. Younkin, A. G. Mills, J. Lokvam, K. G. Dexter, R. T. Pennington, C. A. Kidner, J. A. Nicholls, O. Loiseau, T. A. Kursar, P. D. Coley, Diversity and divergence: Evolution of secondary metabolism in the tropical tree genus *Inga*. *New Phytol.* **237**, 631–642 (2023).

54. R. M. LoPachin, D. S. Barber, T. Gavin, Molecular mechanisms of the conjugated α,β-unsaturated carbonyl derivatives: Relevance to neurotoxicity and neurodegenerative diseases. *Toxicol. Sci.* **104**, 235–249 (2008).

55. J. Widomska, W. K. Subczynski, Mechanisms enhancing the protective functions of macular xanthophylls in the retina during oxidative stress. *Exp. Eye Res.* **178**, 238–246 (2019).

56. M. H. Tran, D.-P. Phan, E. Y. Lee, Review on lignin modifications toward natural UV protection ingredient for lignin-based sunscreens. *Green Chem.* **23**, 4633–4646 (2021).

57. N.-H. Tan, J. Zhou, Plant cyclopeptides. *Chem. Rev.* **106**, 840–895 (2006).

58. G.-H. Lim, R. Singhal, A. Kachroo, P. Kachroo, Fatty acid- and lipid-mediated signaling in plant defense. *Annu. Rev. Phytopathol.* **55**, 505–536 (2017).

59. T. H. Yeats, J. K. C. Rose, The formation and function of plant cuticles. *Plant Physiol.* **163**, 5–20 (2013).

60. Q. Liu, L. Luo, L. Zheng, Lignins: Biosynthesis and biological functions in plants. *Int. J. Mol. Sci.* **19**, 335 (2018).

61. P. E. Staswick, Storage proteins of vegetative plant tissues. *Annu. Rev. Plant. Physiol. Plant. Mol. Biol.* **45**, 303–322 (1994).

62. T. O. Sandberg, C. Weinberger, J.-H. Smått, Molecular dynamics on wood-derived lignans analyzed by intermolecular network theory. *Molecules* **23**, 1990 (2018).

63. M. Pollard, F. Beisson, Y. Li, J. B. Ohlrogge, Building lipid barriers: Biosynthesis of cutin and suberin. *Trends Plant Sci.* **13**, 236–246 (2008).

64. R. Salguero-Gómez, O. R. Jones, E. Jongejans, S. P. Blomberg, D. J. Hodgson, C. Mbeau-Ache, P. A. Zuidema, H. de Kroon, Y. M. Buckley, Fast-slow continuum and reproductive strategies structure plant life-history variation worldwide. *Proc. Natl. Acad. Sci. U.S.A.* **113**, 230–235 (2016).

65. P. D. Coley, in *Plant-Animal Interactions: Evolutionary Ecology in Tropical and Temperate Regions*, P. W. Price, T. M. Lewison, G. W. Fernandes, W. W. Benson, Eds. (John Wiley and Sons, 1991), pp. 54–69.

66. R. Sulpice, P. C. McKeown, Moving toward a comprehensive map of central plant metabolism. *Annu. Rev. Plant Biol.* **66**, 187–210 (2015).

67. S. B. Powles, Photoinhibition of photosynthesis induced by visible light. *Annu. Rev. Plant Physiol.* **35**, 15–44 (1984).

68. J. Berry, O. Bjorkman, Photosynthetic response and adaptation to temperature in higher plants. *Annu. Rev. Plant Physiol.* **31**, 491–543 (1980).

69. B. David, F. Aussiel, in *Handbook of Chemical and Biological Plant Analytical Methods*, K. Hostettmann, Ed. (Wiley, Chichester, West Sussex, ed. 1, 2014), p. 1176.

70. B. David, F. Ausseil, in *Encyclopedia of analytical chemistry: Applications, theory and instrumentation*, R. A. Meyers, Ed. (John Wiley & Sons, 2006), pp. 1–24.

71. T. Pluskal, S. Castillo, A. Villar-Briones, M. Oresic, MZmine 2: Modular framework for processing, visualizing, and analyzing mass spectrometry-based molecular profile data. *BMC Bioinformatics* **11**, 395 (2010).

72. O. D. Myers, S. J. Sumner, S. Li, S. Barnes, X. Du, One step forward for reducing false positive and false negative compound identifications from mass spectrometry metabolomics data: New algorithms for constructing extracted ion chromatograms and detecting chromatographic peaks. *Anal. Chem.* **89**, 8696–8703 (2017).

73. H. Tsugawa, T. Cajka, T. Kind, Y. Ma, B. Higgins, K. Ikeda, M. Kanazawa, J. VanderGheynst, O. Fiehn, M. Arita, MS-DIAL: Data-independent MS/MS deconvolution for comprehensive metabolome analysis. *Nat. Methods* **12**, 523–526 (2015).

74. A. M. Frank, N. Bandeira, Z. Shen, S. Tanner, S. P. Briggs, R. D. Smith, P. A. Pevzner, Clustering millions of tandem mass spectra. *J. Proteome Res.* **7**, 113–122 (2008).

75. M. Wang, J. J. Carver, V. V. Phelan, L. M. Sanchez, N. Garg, Y. Peng, D. D. Nguyen, J. Watrous, C. A. Kapono, T. Luzzatto-Knaan, C. Porto, A. Bouslimani, A. V. Melnik, M. J. Meehan, W.-T. Liu, M. Crüsemann, P. D. Boudreau, E. Esquenazi, M. Sandoval-Calderón, R. D. Kersten, L. A. Pace, R. A. Quinn, K. R. Duncan, C.-C. Hsu, D. J. Floros, R. G. Gavilan, K. Kleigrewe, T. Northen, R. J. Dutton, D. Parrot, E. E. Carlson, B. Aigle, C. F. Michelsen, L. Jelsbak, C. Sohlenkamp, P. Pevzner, A. Edlund, J. McLean, J. Piel, B. T. Murphy, L. Gerwick, C.-C. Liaw, Y.-L. Yang, H.-U. Humpf, M. Maansson, R. A. Keyzers, A. C. Sims, A. R. Johnson, A. M. Sidebottom, B. E. Sedio, A. Klitgaard, C. B. Larson, C. A. Boya, D. Torres-Mendoza, D. J. Gonzalez, D. B. Silva, L. M. Marques, D. P. Demarque, E. Pociute, E. C. O'Neill, E. Briand, E. J. N. Helfrich, E. A. Granatosky, E. Glukhov, F. Ryffel, H. Houson, H. Mohimani, J. J. Kharbush, Y. Zeng, J. A. Vorholt, K. L. Kurita, P. Charusanti, K. L. McPhail, K. F. Nielsen, L. Vuong, M. Elfeki, M. F. Traxler, N. Engene, N. Koyama, O. B. Vining, R. Baric, R. R. Silva, S. J. Mascuch, S. Tomasi, S. Jenkins, V. Macherla, T. Hoffman, V. Agarwal, P. G. Williams, J. Dai, R. Neupane, J. Gurr, A. M. C. Rodríguez, A. Lamsa, C. Zhang, K. Dorrestein, B. M. Duggan, J.

Almaliti, N. Bandeira, Sharing and community curation of mass spectrometry data with GNPS. *Nat. Biotechnol.* **34**, 828–837 (2016).

76. P.-M. Allard, T. Péresse, J. Bisson, K. Gindro, L. Marcourt, V. C. Pham, F. Roussi, M. Litaudon, J.-L. Wolfender, Integration of molecular networking and in-silico MS/MS fragmentation for natural products dereplication. *Anal. Chem.* **88**, 3317–3323 (2016).

77. A. Rutz, M. Dounoue-Kubo, S. Ollivier, J. Bisson, M. Bagheri, T. Saesong, S. N. Ebrahimi, K. Ingkaninan, J.-L. Wolfender, P.-M. Allard, Taxonomically informed scoring enhances confidence in natural products annotation. *Front. Plant Sci.* **10**, 1329 (2019).

78. A. Rutz, J. Bisson, P.-M. Allard, The LOTUS initiative for open natural products research: Frozen dataset union wikidata (with metadata) (Zenodo, 2021); https://doi.org/10.5281/zenodo.5794107.

79. P.-M. Allard, J. Bisson, A. Rutz, ISDB: In silico spectral databases of natural products (Zenodo, 2021), https://doi.org/10.5281/zenodo.5607264.

80. A. Rutz, M. Sorokina, J. Galgonek, D. Mietchen, E. Willighagen, A. Gaudry, J. G. Graham, R. Stephan, R. Page, J. Vondrášek, C. Steinbeck, G. F. Pauli, J.-L. Wolfender, J. Bisson, P.-M. Allard, The LOTUS initiative for open knowledge management in natural products research. *eLife* **11**, e70780 (2022).

81. R. Guha, Chemical informatics functionality in *R. J. Stat. Softw.* **18**, 1–16 (2007).

82. R Core Team, *R: A language and environment for statistical computing* (R Foundation for Statistical Computing, 2014).

83. C. O. Wilke, *cowplot: Streamlined Plot Theme and Plot Annotations for "ggplot2"* (R Project, 2019).

84. M. Dowle, A. Srinivasan, *data.table: Extension of `data.frame`* (R Project, 2021).

85. W. Michael Landau, The drake R package: a pipeline toolkit for reproducibility and high-performance computing. *JOSS* **3**, 550 (2018).

86. D. Vaughan, M. Dancho, *furrr: Apply Mapping Functions in Parallel using Futures* (R Project, 2021).

87. H. Bengtsson, A unifying framework for parallel and distributed processing in R using futures. arXiv:2008.00553 [cs.DC] (2021).

88. W. R. Revelle, *psych: Procedures for Personality and Psychological Research* (Northwestern University, 2019).

89. H. Wickham, M. Averick, J. Bryan, W. Chang, L. McGowan, R. François, G. Grolemund, A. Hayes, L. Henry, J. Hester, M. Kuhn, T. Pedersen, E. Miller, S. Bache, K. Müller, J. Ooms, D. Robinson, D. Seidel, V. Spinu, K. Takahashi, D. Vaughan, C. Wilke, K. Woo, H. Yutani, Welcome to the tidyverse. *JOSS* **4**, 1686 (2019).

90. S. A. Chamberlain, E. Szöcs, taxize: Taxonomic search and retrieval in R. *F1000Res.* **2**, 191 (2013).

91. W. Cornwell, R. FitzJohn, M. Pennell, *taxonlookup: A dynamically-updating versioned taxonomic resource for vascular plants* (R Project, 2016).

92. J. Kattge, G. Bönisch, S. Díaz, S. Lavorel, I. C. Prentice, P. Leadley, S. Tautenhahn, G. D. A. Werner, T. Aakala, M. Abedi, A. T. R. Acosta, G. C. Adamidis, K. Adamson, M. Aiba, C. H. Albert, J. M. Alcántara, Carolina Alcázar C, I. Aleixo, H. Ali, B. Amiaud, C. Ammer, M. M. Amoroso, M. Anand, C. Anderson, N. Anten, J. Antos, D. M. G. Apgaua, T.-L. Ashman, D. H. Asmara, G. P. Asner, M. Aspinwall, O. Atkin, I. Aubin, L. Baastrup-Spohr, K. Bahalkeh, M. Bahn, T. Baker, W. J. Baker, J. P. Bakker, D. Baldocchi, J. Baltzer, A. Banerjee, A. Baranger, J. Barlow, D. R. Barneche, Z. Baruch, D. Bastianelli, J. Battles, W. Bauerle, M. Bauters, E. Bazzato, M. Beckmann, H. Beeckman, C. Beierkuhnlein, R. Bekker, G. Belfry, M. Belluau, M. Beloiu, R. Benavides, L. Benomar, Mary Lee Berdugo-Lattke, E. Berenguer, R. Bergamin, J. Bergmann, M. B. Carlucci, L. Berner, M. Bernhardt-Römermann, C. Bigler, A. D. Bjorkman, C. Blackman, C. Blanco, B. Blonder, D. Blumenthal, K. T. Bocanegra-González, P. Boeckx, S. Bohlman, K. Böhning-Gaese, L. Boisvert-Marsh, W. Bond, B. Bond-Lamberty, A. Boom, C. C. F. Boonman, K. Bordin, E. H. Boughton, V. Boukili, David M J S Bowman, S. Bravo, M. R. Brendel, M. R. Broadley, K. A. Brown, H. Bruelheide, F. Brumnich, H. H. Bruun, D. Bruy, S. W. Buchanan, S. F. Bucher, N. Buchmann, R. Buitenwerf, D. E. Bunker, J. Bürger, S. Burrascano, D. F. R. P. Burslem, B. J. Butterfield, C. Byun, M. Marques, M. C. Scalon, M. Caccianiga, M. Cadotte, M. Cailleret, J. Camac, J. J. Camarero, C. Campany, G. Campetella, J. A. Campos, L. Cano-Arboleda, R. Canullo, M. Carbognani, F. Carvalho, F. Casanoves, B. Castagneyrol, J.

A. Catford, J. Cavender-Bares, B. E. L. Cerabolini, M. Cervellini, E. Chacón-Madrigal, K. Chapin, F. S. Chapin, S. Chelli, S.-C. Chen, A. Chen, P. Cherubini, F. Chianucci, B. Choat, K.-S. Chung, M. Chytrý, D. Ciccarelli, L. Coll, C. G. Collins, L. Conti, D. Coomes, J. H. C. Cornelissen, W. K. Cornwell, P. Corona, M. Coyea, J. Craine, D. Craven, J. P. G. M. Cromsigt, A. Csecserits, K. Cufar, M. Cuntz, A. C. da Silva, K. M. Dahlin, M. Dainese, I. Dalke, M. D. Fratte, A. T. Dang-Le, J. Danihelka, M. Dannoura, S. Dawson, A. J. de Beer, A. De Frutos, J. R. De Long, B. Dechant, S. Delagrange, N. Delpierre, G. Derroire, A. S. Dias, M. H. Diaz-Toribio, P. G. Dimitrakopoulos, M. Dobrowolski, D. Doktor, P. Dřevojan, N. Dong, J. Dransfield, S. Dressler, L. Duarte, E. Ducouret, S. Dullinger, W. Durka, R. Duursma, O. Dymova, A. E.-Vojtkó, R. L. Eckstein, H. Ejtehadi, J. Elser, T. Emilio, K. Engemann, M. B. Erfanian, A. Erfmeier, A. Esquivel-Muelbert, G. Esser, M. Estiarte, T. F. Domingues, W. F. Fagan, J. Fagúndez, D. S. Falster, Y. Fan, J. Fang, E. Farris, F. Fazlioglu, Y. Feng, F. Fernandez-Mendez, C. Ferrara, J. Ferreira, A. Fidelis, B. Finegan, J. Firn, T. J. Flowers, D. F. B. Flynn, V. Fontana, E. Forey, C. Forgiarini, L. François, M. Frangipani, D. Frank, C. Frenette-Dussault, G. T. Freschet, E. L. Fry, N. M. Fyllas, G. G. Mazzochini, S. Gachet, R. Gallagher, G. Ganade, F. Ganga, P. García-Palacios, V. Gargaglione, E. Garnier, J. L. Garrido, A. L. de Gasper, G. Gea-Izquierdo, D. Gibson, A. N. Gillison, A. Giroldo, M.-C. Glasenhardt, S. Gleason, M. Gliesch, E. Goldberg, B. Göldel, E. Gonzalez-Akre, J. L. Gonzalez-Andujar, A. González-Melo, A. González-Robles, B. J. Graae, E. Granda, S. Graves, W. A. Green, T. Gregor, N. Gross, G. R. Guerin, A. Günther, A. G. Gutiérrez, L. Haddock, A. Haines, J. Hall, A. Hambuckers, W. Han, S. P. Harrison, W. Hattingh, J. E. Hawes, T. He, P. He, J. M. Heberling, A. Helm, S. Hempel, J. Hentschel, B. Hérault, A.-M. Hereş, K. Herz, M. Heuertz, T. Hickler, P. Hietz, P. Higuchi, A. L. Hipp, A. Hirons, M. Hock, J. A. Hogan, K. Holl, O. Honnay, D. Hornstein, E. Hou, N. Hough-Snee, K. A. Hovstad, T. Ichie, B. Igić, E. Illa, M. Isaac, M. Ishihara, L. Ivanov, L. Ivanova, C. M. Iversen, J. Izquierdo, R. B. Jackson, B. Jackson, H. Jactel, A. M. Jagodzinski, U. Jandt, S. Jansen, T. Jenkins, A. Jentsch, J. R. P. Jespersen, G.-F. Jiang, J. L. Johansen, D. Johnson, E. J. Jokela, C. A. Joly, G. J. Jordan, G. S. Joseph, D. Junaedi, R. R. Junker, E. Justes, R. Kabzems, J. Kane, Z. Kaplan, T. Kattenborn, L. Kavelenova, E. Kearsley, A. Kempel, T. Kenzo, A. Kerkhoff, M. I. Khalil, N. L. Kinlock, W. D. Kissling, K. Kitajima, T. Kitzberger, R. Kjøller, T. Klein, M. Kleyer, J. Klimešová, J. Klipel, B. Kloeppel, S. Klotz, J. M. H. Knops, T. Kohyama, F. Koike, J. Kollmann, B. Komac, K. Komatsu, C. König, N. J. B. Kraft, K. Kramer, H. Kreft, I. Kühn, D. Kumarathunge, J. Kuppler, H. Kurokawa, Y. Kurosawa, S. Kuyah, J.-P. Laclau, B. Lafleur, E. Lallai, E. Lamb, A. Lamprecht, D. J. Larkin, D. Laughlin, Y. Le Bagousse-Pinguet, G. le Maire, P. C le Roux, E. le Roux, T. Lee, F. Lens, S.

L. Lewis, B. Lhotsky, Y. Li, X. Li, J. W. Lichstein, M. Liebergesell, J. Y. Lim, Y.-S. Lin, J. C. Linares, C. Liu, D. Liu, U. Liu, S. Livingstone, J. Llusià, M. Lohbeck, Á. López-García, G. Lopez-Gonzalez, Z. Lososová, F. Louault, B. A. Lukács, P. Lukeš, Y. Luo, M. Lussu, S. Ma, C. M. R. Pereira, M. Mack, V. Maire, A. Mäkelä, H. Mäkinen, A. C. M. Malhado, A. Mallik, P. Manning, S. Manzoni, Z. Marchetti, L. Marchino, V. Marcilio-Silva, E. Marcon, M. Marignani, L. Markesteijn, A. Martin, C. Martínez-Garza, J. Martínez-Vilalta, T. Mašková, K. Mason, N. Mason, T. J. Massad, J. Masse, I. Mayrose, J. M. Carthy, M Luke Mc Cormack, K. M. Culloh, I. R. Mc Fadden, B. J. Mc Gill, M. Y. Mc Partland, J. S. Medeiros, B. Medlyn, P. Meerts, Z. Mehrabi, P. Meir, F. P. L. Melo, M. Mencuccini, C. Meredieu, J. Messier, I. Mészáros, J. Metsaranta, S. T. Michaletz, C. Michelaki, S. Migalina, R. Milla, J. E. D. Miller, V. Minden, R. Ming, K. Mokany, A. T. Moles, A. Molnárth, J. Molofsky, M. Molz, R. A. Montgomery, A. Monty, L. Moravcová, A. Moreno-Martínez, M. Moretti, A. S. Mori, S. Mori, D. Morris, J. Morrison, L. Mucina, S. Mueller, C. D. Muir, S. C. Müller, F. Munoz, I. H. Myers-Smith, R. W. Myster, M. Nagano, S. Naidu, A. Narayanan, B. Natesan, L. Negoita, A. S. Nelson, E. L. Neuschulz, J. Ni, G. Niedrist, J. Nieto, Ü. Niinemets, R. Nolan, H. Nottebrock, Y. Nouvellon, A. Novakovskiy; Nutrient Network; K. O. Nystuen, A. O'Grady, K. O'Hara, A. O'Reilly-Nugent, S. Oakley, W. Oberhuber, T. Ohtsuka, R. Oliveira, K. Öllerer, M. E. Olson, V. Onipchenko, Y. Onoda, R. E. Onstein, J. C. Ordonez, N. Osada, I. Ostonen, G. Ottaviani, S. Otto, G. E. Overbeck, W. A. Ozinga, A. T. Pahl, C. E. T. Paine, R. J. Pakeman, A. C. Papageorgiou, E. Parfionova, M. Pärtel, M. Patacca, S. Paula, J. Paule, H. Pauli, J. G. Pausas, B. Peco, J. Penuelas, A. Perea, P. L. Peri, A. C. Petisco-Souza, A. Petraglia, A. M. Petritan, O. L. Phillips, S. Pierce, V. D. Pillar, J. Pisek, A. Pomogaybin, H. Poorter, A. Portsmuth, P. Poschlod, C. Potvin, D. Pounds, A. S. Powell, S. A. Power, A. Prinzing, G. Puglielli, P. Pyšek, V. Raevel, A. Rammig, J. Ransijn, C. A. Ray, P. B. Reich, M. Reichstein, D. E. B. Reid, M. Réjou-Méchain, V. R. de Dios, S. Ribeiro, S. Richardson, K. Riibak, M. C. Rillig, F. Riviera, E. M. R. Robert, S. Roberts, B. Robroek, A. Roddy, A. V. Rodrigues, A. Rogers, E. Rollinson, V. Rolo, C. Römermann, D. Ronzhina, C. Roscher, J. A. Rosell, M. F. Rosenfield, C. Rossi, D. B. Roy, S. Royer-Tardif, N. Rüger, R. Ruiz-Peinado, S. B. Rumpf, G. M. Rusch, M. Ryo, L. Sack, A. Saldaña, B. Salgado-Negret, R. Salguero-Gomez, I. Santa-Regina, A. C. Santacruz-García, J. Santos, J. Sardans, B. Schamp, M. Scherer-Lorenzen, M. Schleuning, B. Schmid, M. Schmidt, S. Schmitt, J. V. Schneider, S. D. Schowanek, J. Schrader, F. Schrodt, B. Schuldt, F. Schurr, G. S. Garvizu, M. Semchenko, C. Seymour, J. C. Sfair, J. M. Sharpe, C. S. Sheppard, S. Sheremetiev, S. Shiodera, B. Shipley, T. A. Shovon, A. Siebenkäs, C. Sierra, V. Silva, M. Silva, T. Sitzia, H. Sjöman, M. Slot, N. G. Smith, D. Sodhi, P. Soltis, D. Soltis, B.

Somers, G. Sonnier, M. V. Sørensen, E. E. Sosinski Jr, N. A. Soudzilovskaia, A. F. Souza, M. Spasojevic, M. G. Sperandii, A. B. Stan, J. Stegen, K. Steinbauer, J. G. Stephan, F. Sterck, D. B. Stojanovic, T. Strydom, M. L. Suarez, J.-C. Svenning, I. Svitková, M. Svitok, M. Svoboda, E. Swaine, N. Swenson, M. Tabarelli, K. Takagi, U. Tappeiner, R. Tarifa, S. Tauugourdeau, C. Tavsanoglu, M. T. Beest, L. Tedersoo, N. Thiffault, D. Thom, E. Thomas, K. Thompson, P. E. Thornton, W. Thuiller, L. Tichý, D. Tissue, M. G. Tjoelker, D. Y. P. Tng, J. Tobias, P. Török, T. Tarin, J. M. Torres-Ruiz, B. Tóthmérész, M. Treurnicht, V. Trivellone, F. Trolliet, V. Trotsiuk, J. L. Tsakalos, I. Tsiripidis, N. Tysklind, T. Umehara, V. Usoltsev, M. Vadeboncoeur, J. Vaezi, F. Valladares, J. Vamosi, P. M. van Bodegom, M. van Breugel, E. Van Cleemput, M. van de Weg, S. van der Merwe, F. van der Plas, M. T. van der Sande, M. van Kleunen, K. Van Meerbeek, M. Vanderwel, K. A. Vanselow, A. Vårhammar, L. Varone, M. Y. V. Valderrama, K. Vassilev, M. Vellend, E. J. Veneklaas, H. Verbeeck, K. Verheyen, A. Vibrans, I. Vieira, J. Villacís, C. Violle, P. Vivek, K. Wagner, M. Waldram, A. Waldron, A. P. Walker, M. Waller, G. Walther, H. Wang, F. Wang, W. Wang, H. Watkins, J. Watkins, U. Weber, J. T. Weedon, L. Wei, P. Weigelt, E. Weiher, A. W. Wells, C. Wellstein, E. Wenk, M. Westoby, A. Westwood, P. J. White, M. Whitten, M. Williams, D. E. Winkler, K. Winter, C. Womack, I. J. Wright, S. J. Wright, J. Wright, B. X. Pinho, F. Ximenes, T. Yamada, K. Yamaji, R. Yanai, N. Yankov, B. Yguel, K. J. Zanini, A. E. Zanne, D. Zelený, Y.-P. Zhao, J. Zheng, J. Zheng, K. Ziemińska, C. R. Zirbel, G. Zizka, I. C. Zo-Bi, G. Zotz, C. Wirth, TRY plant trait database - enhanced coverage and open access. *Glob. Chang. Biol.* **26**, 119–188 (2020).

93. J. P. Grime, Vegetation classification by reference to strategies. *Nature* **250**, 26–31 (1974).

94. F. Schrodt, J. Kattge, H. Shan, F. Fazayeli, J. Joswig, A. Banerjee, M. Reichstein, G. Bönisch, S. Díaz, J. Dickie, A. Gillison, A. Karpatne, S. Lavorel, P. Leadley, C. B. Wirth, I. J. Wright, S. J. Wright, P. B. Reich, BHPMF - a hierarchical Bayesian approach to gap-filling and trait prediction for macroecology and functional biogeography. *Glob. Ecol. Biogeogr.* **24**, 1510–1521 (2015).

95. S. van Buuren, K. Groothuis-Oudshoorn, mice: Multivariate imputation by chained equations in *R. J. Stat. Softw.* **45**, 1–67 (2011).

96. C. Maldonado, C. I. Molina, A. Zizka, C. Persson, C. M. Taylor, J. Albán, E. Chilquillo, N. Rønsted, A. Antonelli, Estimating species diversity and distribution in the era of big data: To what extent can we trust public databases? *Glob. Ecol. Biogeogr.* **24**, 973–984 (2015).

97. A. Zizka, D. Silvestro, T. Andermann, J. Azevedo, C. Duarte Ritter, D. Edler, H. Farooq, A. Herdean, M. Ariza, R. Scharn, S. Svanteson, N. Wengström, V. Zizka, A. Antonelli, CoordinateCleaner: Standardized cleaning of occurrence records from biological collection databases. *Methods Ecol. Evol.* **10**, 744–751 (2019).

98. D. S. Maynard, L. Bialic-Murphy, C. M. Zohner, C. Averill, J. van den Hoogen, H. Ma, L. Mo, G. R. Smith, A. T. R. Acosta, I. Aubin, E. Berenguer, C. C. F. Boonman, J. A. Catford, B. E. L. Cerabolini, A. S. Dias, A. González-Melo, P. Hietz, C. H. Lusk, A. S. Mori, Ü. Niinemets, V. D. Pillar, B. X. Pinho, J. A. Rosell, F. M. Schurr, S. N. Sheremetev, A. C. da Silva, Ê. Sosinski, P. M. van Bodegom, E. Weiher, G. Bönisch, J. Kattge, T. W. Crowther, Global relationships in tree functional traits. *Nat. Commun.* **13**, 3185 (2022).

99. J. Oksanen, F. G. Blanchet, M. Friendly, R. Kindt, P. Legendre, D. McGlinn, P. R. Minchin, R. B. O'Hara, G. L. Simpson, P. Solymos, M. H. H. Stevens, E. Szoecs, H. Wagner, *vegan: Community Ecology Package* (R Project, 2019).

100. W. Weckwerth, Metabolomics in systems biology. *Annu. Rev. Plant Biol.* **54**, 669–689 (2003).

101. H. Uthe, N. M. van Dam, M. R. Hervé, M. Sorokina, K. Peters, A. Weinhold, in *Plant Metabolomics in full swing* (Elsevier, 2021), vol. 98 of *Advances in botanical research*, pp. 163–203.

102. K. Habel, R. Grasman, R. B. Gramacy, P. Mozharovskyi, D. C. Sterrat, *geometry: Mesh Generation and Surface Tessellation* (R, 2022).

103. S. Dray, A.-B. Dufour, The ade4 package: Implementing the duality diagram for ecologists. *J. Stat. Softw.* **22**, 1–20 (2007).

104. R. Salguero-Gómez, Applications of the fast-slow continuum and reproductive strategy framework of plant life histories. *New Phytol.* **213**, 1618–1624 (2017).

105. H. Kusano, H. Li, H. Minami, Y. Kato, H. Tabata, K. Yazaki, Evolutionary developments in plant specialized metabolism, exemplified by two transferase families. *Front. Plant Sci.* **10**, 794 (2019).

106. R. T. Bush, F. A. McInerney, Leaf wax n-alkane distributions in and across modern plants: Implications for paleoecology and chemotaxonomy. *Geochim. Cosmochim. Acta* **117**, 161–179 (2013).

107. L. F. Maia, V. E. De Oliveira, H. G. M. Edwards, L. F. C. De Oliveira, The diversity of linear conjugated polyenes and colours in nature: Raman spectroscopy as a diagnostic tool. *ChemPhysChem* **22**, 231–249 (2021).

108. J. K. Hoober, L. L. Eggink, M. Chen, Chlorophylls, ligands and assembly of light-harvesting complexes in chloroplasts. *Photosyn. Res.* **94**, 387–400 (2007).

109. D. F. Veber, S. R. Johnson, H.-Y. Cheng, B. R. Smith, K. W. Ward, K. D. Kopple, Molecular properties that influence the oral bioavailability of drug candidates. *J. Med. Chem.* **45**, 2615–2623 (2002).

110. P. Ertl, B. Rohde, P. Selzer, Fast calculation of molecular polar surface area as a sum of fragment-based contributions and its application to the prediction of drug transport properties. *J. Med. Chem.* **43**, 3714–3717 (2000).

111. K. Palm, P. Stenberg, K. Luthman, P. Artursson, Polar molecular surface properties predict the intestinal absorption of drugs in humans. *Pharm. Res.* **14**, 568–571 (1997).

112. G. A. Jeffrey, W. Saenger, *Hydrogen bonding in biological structures* (Springer Berlin Heidelberg, 1991).
